# New York City

**Published:** 1896-11

**Authors:** 


*New York City*, from January 1, to August 31, 1896, received 53,755,433 gallons of milk; of cream and condensed milk 2,283,130 gallons.

